# 2,4-Dihydr­oxy-*N*′-(2-hydr­oxy-4-methoxy­benzyl­idene)benzohydrazide

**DOI:** 10.1107/S1600536810012420

**Published:** 2010-04-10

**Authors:** You-Yue Han, Qiu-Rong Zhao

**Affiliations:** a Department of Chemistry and Life Sciences, Chuzhou University, Chuzhou, Anhui 239000, People’s Republic of China

## Abstract

In the title compound, C_15_H_14_N_2_O_5_, the dihedral angle between the two benzene rings is 4.3 (3)° and the mol­ecule adopts an *E* configuration with respect to the C=N bond. Intra­molecular O—H⋯N and N—H⋯O hydrogen bonds are observed. In the crystal structure, the mol­ecules are linked through inter­molecular N—H⋯O and O—H⋯O hydrogen bonds to form layers parallel to the *ac* plane.

## Related literature

For the biological properties of hydrazone compounds, see: Patil *et al.* (2010 ▶); Cukurovali *et al.* (2006 ▶). For bond-length data, see: Allen *et al.* (1987 ▶). For related structures, see: Mohd Lair *et al.* (2009 ▶); Lin & Sang (2009 ▶); Suleiman Gwaram *et al.* (2010 ▶); Li & Ban (2009 ▶); Lo & Ng (2009 ▶); Ning & Xu (2009 ▶); Zhu *et al.* (2009 ▶).
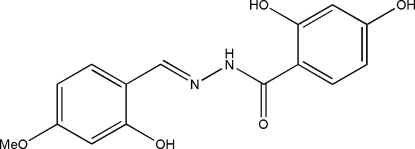


## Experimental

### 

#### Crystal data

C_15_H_14_N_2_O_5_
*M* *_r_* = 302.28
Monoclinic, 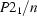

*a* = 10.560 (3) Å
*b* = 12.752 (3) Å
*c* = 11.313 (2) Å
β = 112.853 (3)°
*V* = 1403.8 (6) Å^3^
*Z* = 4
Mo *K*α radiation
μ = 0.11 mm^−1^
*T* = 298 K
0.30 × 0.27 × 0.25 mm

#### Data collection

Bruker SMART CCD area-detector diffractometer
Absorption correction: multi-scan (*SADABS*; Bruker, 2001 ▶) *T*_min_ = 0.968, *T*_max_ = 0.973
7972 measured reflections
3030 independent reflections
1023 reflections with *I* > 2σ(*I*)
*R*_int_ = 0.127

#### Refinement

*R*[*F* ^2^ > 2σ(*F* ^2^)] = 0.054
*wR*(*F* ^2^) = 0.182
*S* = 0.74
3030 reflections
206 parameters
1 restraint
H atoms treated by a mixture of independent and constrained refinement
Δρ_max_ = 0.24 e Å^−3^
Δρ_min_ = −0.28 e Å^−3^

### 

Data collection: *SMART* (Bruker, 2007 ▶); cell refinement: *SAINT* (Bruker, 2007 ▶); data reduction: *SAINT*; program(s) used to solve structure: *SHELXTL* (Sheldrick, 2008 ▶); program(s) used to refine structure: *SHELXTL*; molecular graphics: *SHELXTL*; software used to prepare material for publication: *SHELXTL*.

## Supplementary Material

Crystal structure: contains datablocks global, I. DOI: 10.1107/S1600536810012420/ci5074sup1.cif

Structure factors: contains datablocks I. DOI: 10.1107/S1600536810012420/ci5074Isup2.hkl

Additional supplementary materials:  crystallographic information; 3D view; checkCIF report

## Figures and Tables

**Table 1:** Hydrogen-bond geometry (Å, °)

*D*—H⋯*A*	*D*—H	H⋯*A*	*D*⋯*A*	*D*—H⋯*A*
O1—H1⋯N1	0.82	1.92	2.635 (3)	145
N2—H2⋯O4	0.91 (1)	2.03 (3)	2.670 (3)	126 (3)
N2—H2⋯O1^i^	0.91 (1)	2.43 (2)	3.240 (4)	149 (3)
O5—H5⋯O2^ii^	0.82	2.05	2.865 (4)	172
O4—H4⋯O3^i^	0.82	1.79	2.607 (3)	174

Symmetry codes: (i) 
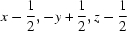
; (ii) 
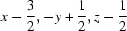
.

**Fig. 1.**
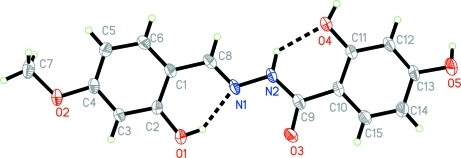
The molecular structure of the title compound with atom labels and 30% probability displacement ellipsoids for non-H atoms. Intramolecular hydrogen bonds are shown as dashed lines.

**Fig. 2.**
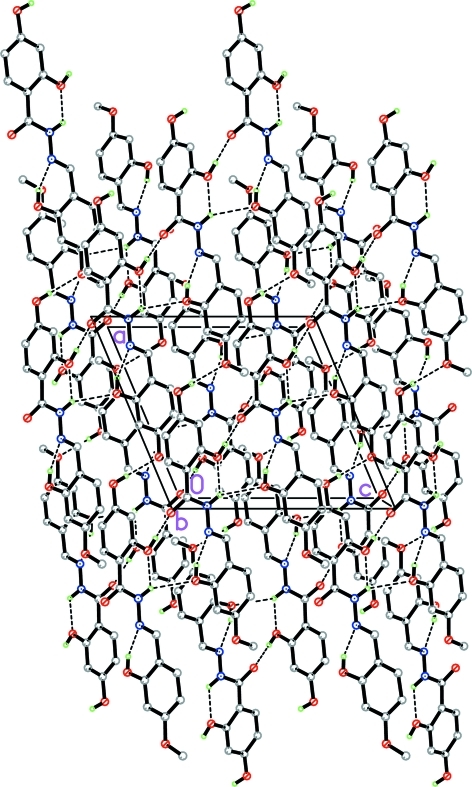
The molecular packing of the title compound, viewed along the b axis. Hydrogen bonds are shown as dashed lines.

## supplementary crystallographic information

### Comment

Hydrazone compounds have been widely investigated for their biological properties (Patil *et al.*, 2010; Cukurovali *et al.*, 2006). Furthermore, the crystal structures of hydrazone compounds have also attracted much attention in recent years (Mohd Lair *et al.*, 2009; Lin & Sang, 2009; Suleiman Gwaram *et al.*, 2010). In the present work, the title new hydrazone compound is reported.

In the molecule of the title compound (Fig. 1), the dihedral angle between the two benzene rings is 4.3 (3)°. The molecule adopts an *E* configuration with respect to the C═N bond. There are intramolecular O–H···N and N–H···O hydrogen bonds (Table 1) in the molecule. All the bond lengths are within normal ranges (Allen *et al.*, 1987) and are comparable with those observed in related structures (Li & Ban, 2009; Lo & Ng, 2009; Ning & Xu, 2009; Zhu *et al.*, 2009).

In the crystal structure, molecules are linked through intermolecular N—H···O and O–H···O hydrogen bonds (Table 1) to form layers parallel to the *ac* plane (Fig. 2).

### Experimental

A mixture of 2-hydroxy-4-methoxybenzaldehyde (0.152 g, 1 mmol) and 2,4-dihydroxybenzohydrazide (0.168 g, 1 mmol) in methanol (50 ml) was stirred at room temperature for 1 h. The mixture was filtered to remove impurities, and then left at room temperature. After a few days, single crystals of the title compound, suitable for X-ray diffraction, were formed.

### Refinement

Atom H2 was located in a difference Fourier map and refined isotropically, with the N–H distance restrained to 0.90 (1) Å. Other H atoms were positioned geometrically and refined using the riding-model approximation, with C–H = 0.93 or 0.96 Å, O–H = 0.82 Å and U_iso_(H) = 1.2U_eq_(C) or U_iso_(H) = 1.5U_eq_(methyl C and O). The ratio of observed to unique reflections is low (34%), and the value of R_int_ is greater (0.127) probably due to the poor diffraction quality of the crystal.

### Crystal data

**Table:**

C_15_H_14_N_2_O_5_	*F*(000) = 632
*M**_r_* = 302.28	*D*_x_ = 1.430 Mg m^−^^3^
Monoclinic, *P*2_1_/*n*	Mo *K*α radiation, λ = 0.71073 Å
Hall symbol: -P 2yn	Cell parameters from 691 reflections
*a* = 10.560 (3) Å	θ = 2.5–24.5°
*b* = 12.752 (3) Å	µ = 0.11 mm^−^^1^
*c* = 11.313 (2) Å	*T* = 298 K
β = 112.853 (3)°	Block, colourless
*V* = 1403.8 (6) Å^3^	0.30 × 0.27 × 0.25 mm
*Z* = 4	

### Data collection

**Table:**

Bruker SMART CCD area-detector diffractometer	3030 independent reflections
Radiation source: fine-focus sealed tube	1023 reflections with *I* > 2σ(*I*)
graphite	*R*_int_ = 0.127
ω scans	θ_max_ = 27.0°, θ_min_ = 2.2°
Absorption correction: multi-scan (*SADABS*; Bruker, 2001)	*h* = −11→13
*T*_min_ = 0.968, *T*_max_ = 0.973	*k* = −16→16
7972 measured reflections	*l* = −14→9

### Refinement

**Table:**

Refinement on *F*^2^	Primary atom site location: structure-invariant direct methods
Least-squares matrix: full	Secondary atom site location: difference Fourier map
*R*[*F*^2^ > 2σ(*F*^2^)] = 0.054	Hydrogen site location: inferred from neighbouring sites
*wR*(*F*^2^) = 0.182	H atoms treated by a mixture of independent and constrained refinement
*S* = 0.74	*w* = 1/[σ^2^(*F*_o_^2^) + (0.0795*P*)^2^] where *P* = (*F*_o_^2^ + 2*F*_c_^2^)/3
3030 reflections	(Δ/σ)_max_ = 0.001
206 parameters	Δρ_max_ = 0.24 e Å^−^^3^
1 restraint	Δρ_min_ = −0.28 e Å^−^^3^

### Special details

**Table:**

Geometry. All esds (except the esd in the dihedral angle between two l.s. planes) are estimated using the full covariance matrix. The cell esds are taken into account individually in the estimation of esds in distances, angles and torsion angles; correlations between esds in cell parameters are only used when they are defined by crystal symmetry. An approximate (isotropic) treatment of cell esds is used for estimating esds involving l.s. planes.
Refinement. Refinement of *F*^2^ against ALL reflections. The weighted *R*-factor wR and goodness of fit *S* are based on *F*^2^, conventional *R*-factors *R* are based on *F*, with *F* set to zero for negative *F*^2^. The threshold expression of *F*^2^ > σ(*F*^2^) is used only for calculating *R*-factors(gt) etc. and is not relevant to the choice of reflections for refinement. *R*-factors based on *F*^2^ are statistically about twice as large as those based on *F*, and *R*- factors based on ALL data will be even larger.

### Fractional atomic coordinates and isotropic or equivalent isotropic displacement parameters (Å^2^)

**Table:**

	*x*	*y*	*z*	*U*_iso_*/*U*_eq_	
N1	0.6400 (2)	0.2494 (2)	0.3907 (3)	0.0457 (8)	
N2	0.4991 (3)	0.2654 (2)	0.3472 (3)	0.0472 (8)	
O1	0.8985 (2)	0.2824 (2)	0.5431 (3)	0.0609 (8)	
H1	0.8162	0.2961	0.5104	0.091*	
O2	1.2425 (2)	0.08925 (18)	0.4832 (2)	0.0563 (7)	
O3	0.5297 (2)	0.36628 (17)	0.5199 (2)	0.0504 (7)	
O4	0.23759 (19)	0.21715 (19)	0.2060 (2)	0.0505 (7)	
H4	0.1705	0.1952	0.1455	0.076*	
O5	−0.1093 (2)	0.4346 (2)	0.2541 (3)	0.0650 (8)	
H5	−0.1518	0.4218	0.1778	0.097*	
C1	0.8268 (3)	0.1629 (3)	0.3636 (3)	0.0391 (9)	
C2	0.9293 (3)	0.2101 (3)	0.4705 (3)	0.0407 (9)	
C3	1.0660 (3)	0.1834 (3)	0.5062 (3)	0.0446 (9)	
H3	1.1327	0.2147	0.5775	0.054*	
C4	1.1035 (3)	0.1099 (3)	0.4356 (3)	0.0432 (9)	
C5	1.0068 (3)	0.0628 (3)	0.3298 (4)	0.0478 (10)	
H5A	1.0325	0.0140	0.2822	0.057*	
C6	0.8696 (3)	0.0899 (3)	0.2955 (4)	0.0492 (10)	
H6	0.8038	0.0580	0.2242	0.059*	
C7	1.2890 (3)	0.0103 (3)	0.4192 (4)	0.0709 (13)	
H7A	1.2419	−0.0543	0.4179	0.106*	
H7B	1.3861	0.0002	0.4639	0.106*	
H7C	1.2700	0.0322	0.3328	0.106*	
C8	0.6814 (3)	0.1853 (3)	0.3265 (3)	0.0435 (9)	
H8	0.6174	0.1524	0.2548	0.052*	
C9	0.4506 (3)	0.3273 (3)	0.4183 (4)	0.0415 (9)	
C10	0.3002 (3)	0.3483 (2)	0.3673 (3)	0.0374 (9)	
C11	0.1983 (3)	0.2962 (3)	0.2647 (3)	0.0370 (8)	
C12	0.0617 (3)	0.3252 (2)	0.2260 (3)	0.0419 (9)	
H12	−0.0052	0.2916	0.1569	0.050*	
C13	0.0249 (3)	0.4037 (3)	0.2898 (4)	0.0437 (9)	
C14	0.1218 (3)	0.4546 (3)	0.3917 (4)	0.0519 (10)	
H14	0.0958	0.5076	0.4343	0.062*	
C15	0.2581 (3)	0.4264 (2)	0.4305 (4)	0.0457 (9)	
H15	0.3235	0.4603	0.5004	0.055*	
H2	0.443 (3)	0.241 (3)	0.2685 (18)	0.080*	

### Atomic displacement parameters (Å^2^)

**Table:**

	*U*^11^	*U*^22^	*U*^33^	*U*^12^	*U*^13^	*U*^23^
N1	0.0189 (15)	0.0627 (19)	0.050 (2)	0.0027 (12)	0.0073 (13)	−0.0013 (16)
N2	0.0205 (15)	0.068 (2)	0.048 (2)	0.0037 (13)	0.0070 (14)	−0.0070 (17)
O1	0.0297 (13)	0.0741 (17)	0.0720 (19)	0.0085 (13)	0.0121 (13)	−0.0242 (16)
O2	0.0291 (13)	0.0700 (17)	0.0685 (19)	0.0128 (11)	0.0176 (12)	−0.0086 (15)
O3	0.0308 (12)	0.0562 (15)	0.0506 (17)	0.0006 (11)	0.0010 (12)	−0.0063 (14)
O4	0.0261 (12)	0.0640 (16)	0.0546 (18)	0.0019 (12)	0.0083 (12)	−0.0129 (14)
O5	0.0297 (13)	0.0760 (18)	0.082 (2)	0.0135 (13)	0.0131 (13)	−0.0088 (17)
C1	0.0244 (17)	0.049 (2)	0.042 (2)	−0.0017 (15)	0.0103 (16)	0.0014 (19)
C2	0.0298 (18)	0.045 (2)	0.049 (2)	0.0039 (16)	0.0172 (17)	−0.0038 (19)
C3	0.0260 (18)	0.057 (2)	0.047 (2)	0.0004 (16)	0.0100 (16)	−0.008 (2)
C4	0.0302 (18)	0.051 (2)	0.054 (2)	0.0027 (16)	0.0217 (18)	0.003 (2)
C5	0.042 (2)	0.051 (2)	0.054 (3)	0.0009 (17)	0.0217 (19)	−0.010 (2)
C6	0.033 (2)	0.058 (2)	0.054 (2)	−0.0082 (17)	0.0142 (18)	−0.010 (2)
C7	0.050 (3)	0.082 (3)	0.085 (3)	0.024 (2)	0.031 (2)	−0.002 (3)
C8	0.0288 (19)	0.053 (2)	0.043 (2)	−0.0066 (15)	0.0084 (17)	0.0018 (19)
C9	0.0279 (18)	0.045 (2)	0.045 (2)	0.0013 (15)	0.0074 (18)	0.0103 (19)
C10	0.0237 (17)	0.044 (2)	0.043 (2)	0.0015 (15)	0.0110 (16)	0.0066 (18)
C11	0.0264 (17)	0.0429 (19)	0.041 (2)	−0.0007 (15)	0.0123 (16)	0.0046 (18)
C12	0.0237 (17)	0.047 (2)	0.049 (2)	−0.0006 (15)	0.0072 (16)	0.0019 (19)
C13	0.0239 (17)	0.048 (2)	0.056 (2)	0.0062 (15)	0.0119 (17)	0.006 (2)
C14	0.040 (2)	0.049 (2)	0.067 (3)	0.0041 (17)	0.021 (2)	−0.008 (2)
C15	0.0331 (19)	0.045 (2)	0.053 (2)	−0.0009 (16)	0.0104 (17)	−0.004 (2)

### Geometric parameters (Å, °)

**Table:**

N1—C8	1.278 (4)	C4—C5	1.374 (4)
N1—N2	1.389 (3)	C5—C6	1.390 (4)
N2—C9	1.362 (4)	C5—H5A	0.93
N2—H2	0.911 (10)	C6—H6	0.93
O1—C2	1.355 (4)	C7—H7A	0.96
O1—H1	0.82	C7—H7B	0.96
O2—C4	1.378 (3)	C7—H7C	0.96
O2—C7	1.434 (4)	C8—H8	0.93
O3—C9	1.232 (4)	C9—C10	1.488 (4)
O4—C11	1.357 (4)	C10—C15	1.397 (4)
O4—H4	0.82	C10—C11	1.406 (4)
O5—C13	1.372 (3)	C11—C12	1.386 (4)
O5—H5	0.82	C12—C13	1.375 (4)
C1—C6	1.390 (4)	C12—H12	0.93
C1—C2	1.407 (4)	C13—C14	1.372 (4)
C1—C8	1.455 (4)	C14—C15	1.380 (4)
C2—C3	1.382 (4)	C14—H14	0.93
C3—C4	1.385 (4)	C15—H15	0.93
C3—H3	0.93		
			
C8—N1—N2	116.6 (3)	H7A—C7—H7B	109.5
C9—N2—N1	118.2 (3)	O2—C7—H7C	109.5
C9—N2—H2	122 (2)	H7A—C7—H7C	109.5
N1—N2—H2	120 (2)	H7B—C7—H7C	109.5
C2—O1—H1	109.5	N1—C8—C1	121.1 (3)
C4—O2—C7	117.3 (3)	N1—C8—H8	119.4
C11—O4—H4	109.5	C1—C8—H8	119.4
C13—O5—H5	109.5	O3—C9—N2	120.7 (3)
C6—C1—C2	117.1 (3)	O3—C9—C10	121.6 (3)
C6—C1—C8	120.3 (3)	N2—C9—C10	117.7 (3)
C2—C1—C8	122.5 (3)	C15—C10—C11	117.8 (3)
O1—C2—C3	117.4 (3)	C15—C10—C9	115.7 (3)
O1—C2—C1	121.7 (3)	C11—C10—C9	126.5 (3)
C3—C2—C1	120.9 (3)	O4—C11—C12	121.5 (3)
C2—C3—C4	119.9 (3)	O4—C11—C10	118.3 (3)
C2—C3—H3	120.0	C12—C11—C10	120.2 (3)
C4—C3—H3	120.0	C13—C12—C11	120.0 (3)
C5—C4—O2	125.1 (3)	C13—C12—H12	120.0
C5—C4—C3	121.0 (3)	C11—C12—H12	120.0
O2—C4—C3	113.8 (3)	O5—C13—C14	117.4 (3)
C4—C5—C6	118.4 (3)	O5—C13—C12	121.6 (3)
C4—C5—H5A	120.8	C14—C13—C12	121.0 (3)
C6—C5—H5A	120.8	C13—C14—C15	119.3 (3)
C5—C6—C1	122.7 (3)	C13—C14—H14	120.4
C5—C6—H6	118.7	C15—C14—H14	120.4
C1—C6—H6	118.7	C14—C15—C10	121.6 (3)
O2—C7—H7A	109.5	C14—C15—H15	119.2
O2—C7—H7B	109.5	C10—C15—H15	119.2

### Hydrogen-bond geometry (Å, °)

**Table:**

*D*—H···*A*	*D*—H	H···*A*	*D*···*A*	*D*—H···*A*
O1—H1···N1	0.82	1.92	2.635 (3)	145
N2—H2···O4	0.91 (1)	2.03 (3)	2.670 (3)	126 (3)
N2—H2···O1^i^	0.91 (1)	2.43 (2)	3.240 (4)	149 (3)
O5—H5···O2^ii^	0.82	2.05	2.865 (4)	172
O4—H4···O3^i^	0.82	1.79	2.607 (3)	174

Symmetry codes: (i) *x*−1/2, −*y*+1/2, *z*−1/2; (ii) *x*−3/2, −*y*+1/2, *z*−1/2.
